# HIV Strategic Information in Non-European Union Countries in the World Health Organization European Region: Capacity Development Needs

**DOI:** 10.2196/publichealth.7357

**Published:** 2017-06-23

**Authors:** Ivana Bozicevic, Senad Handanagic, Jurja Ivana Cakalo, Annemarie Rinder Stengaard, George Rutherford

**Affiliations:** ^1^ WHO Collaborating Centre for HIV Strategic Information University of Zagreb School of Medicine Zagreb Croatia; ^2^ Joint Tuberculosis, HIV/AIDS and Hepatitis Programme, Division of Communicable Diseases, Health Security and Environment WHO Regional Office for Europe Kopenhagen Denmark; ^3^ Global Health Sciences University of California, San Francisco San Francisco, CA United States

**Keywords:** HIV, surveillance, evaluation, Europe, aptitude

## Abstract

**Background:**

Capacity building of the national HIV strategic information system is a core component of the response to the HIV epidemic as it enables understanding of the evolving nature of the epidemic, which is critical for program planning and identification of the gaps and deficiencies in HIV programs.

**Objective:**

The study aims to describe the results of the assessment of the needs for further development of capacities in HIV strategic information systems in the non-European Union (EU) countries in the World Health Organization European Region (EUR).

**Methods:**

Self-administered questionnaires were distributed to national AIDS programs. The first questionnaire was sent to all countries (N=18) to find out, among other issues, the priority level for strengthening a range of HIV surveillance areas and their key gaps and weaknesses. The second questionnaire was sent to 15 countries to more specifically determine capacities for the analysis of the HIV care cascade.

**Results:**

Responses to the first questionnaire were received from 10 countries, whereas 13 countries responded to the second questionnaire. Areas that were most frequently marked as being of high to moderate priority for strengthening were national electronic patient monitoring systems, evaluation of HIV interventions and impact analysis, implementation science, and data analysis. Key weaknesseses were lack of electronic reporting of HIV cases, problems with timeliness and completeness of reporting in HIV cases, under-estimates of the reported number of HIV-related deaths, and limited CD4 count testing at the time of HIV diagnosis. Migrant populations, internally displaced persons, and refugees were most commonly mentioned as groups not covered by surveillance, followed by clients of sex workers and men who have sex with men. The majority of countries reported that they were able to provide the number of people diagnosed with HIV who know their HIV status, which is important for the analysis of cross-sectional and longitudinal HIV care cascades. Ability to report on some of the key impact indicators of HIV programs—viral load suppression and mortality—should be considerably strengthened.

**Conclusions:**

The assessment found a substantial need to invest in surveillance capacities, which is a cornerstone in the development of an evidence-informed response to HIV epidemics.

## Introduction

### Recent Developments in HIV Surveillance

Capacity development is a multidimensional concept with a variety of meanings. Goodman describes capacity as “the ability to carry out stated objectives” [1]. Similarly, Brown and colleagues define capacity development as a process that improves the ability of a person, group, organization, or system to meet its objectives and perform better [2]. The purpose of capacity building in HIV surveillance and monitoring and evaluation (M&E) is to improve the performance of the national HIV surveillance and M&E systems, which primarily means enhancing the ability to produce quality and timely data on the HIV epidemic and the HIV response [3]. Strengthening HIV information systems is the first strategic direction of the World Health Organization (WHO) Global Health Sector Strategy for HIV for 2016-2020 [4]. Using epidemiological and clinical data from these systems, countries should be able to construct cascades of HIV diagnosis, care, and treatment (HIV care cascades) and monitor the processes of reaching the 2020 Joint United Nations Programme on HIV/AIDS (UNAIDS) objectives of 90% of people living with HIV (PLHIV) knowing their HIV status, 90% of PLHIV on antiretroviral treatment (ART), and 90% of people on ART being virally suppressed [5]. The HIV care cascade is a framework for identifying and quantifying the magnitude of the gaps along the continuum of HIV diagnosis, care, and treatment [6,7]. The cascade analysis enables us to identify losses in the continuum of HIV services so that program implementers at facility, regional, or national levels can target resources and interventions more effectively, improve engagement in care for HIV positive individuals, and ultimately prevent new infections. Several data sources and indicators are needed to construct HIV care cascades, including estimates of the number of PLHIV, the number of PLHIV who are diagnosed (obtained from HIV case-based surveillance or surveys), the number of PLHIV who are receiving ART, and the number of

PLHIV who are virally supressed (obtained from HIV patient monitoring systems). The HIV care cascade can be made at the national level but also at sub-national and facility levels and, whenever possible, it should be disaggregated by sex, age groups, and key populations. This enables us to monitor differences in access to services and treatment outcomes across geographical areas and population sub-groups and to improve the coverage and quality of services by providing targeted interventions [8]. The value of the cascade analysis lies in its use as a data and programmatic quality improvement tool. However, the ability to interpret the results of the cascade analysis depends heavily on the availability and the quality of data sources, both of which vary across European countries [9].

### Overview of HIV Epidemics in Eastern Europe and Central Asia

There are several important reasons for the necessity of developing HIV surveillance and monitoring systems in Eastern Europe and Central Asia. The most recent report of the European Centre for Disease Prevention and Control states that the HIV epidemic is still a major concern in Europe, particularly in the eastern part of the WHO European Region [10]. In 2015, 153,407 people were newly diagnosed with HIV in 50 of the 53 countries (no data were available from Bosnia and Herzegovina, Turkmenistan, and Uzbekistan) of the WHO European Region (EUR), which is the highest recorded number of newly diagnosed infections in one year since the start of reporting in the 1980s [10]. According to the UNAIDS estimates, between 2010 and 2015 the number of new HIV infections in Eastern Europe and Central Asia rose by 57%, totaling 190,000 (170,000-200,000) new infections, which is the highest increase globally [11]. In the same period, the number of AIDS-related deaths increased by 22%. HIV treatment coverage among the estimated 1.5 million (1.4 million-1.7 million) PLHIV in Eastern Europe and Central Asia in 2015 was only 21% (20-23%), the second lowest globally after the Middle East and North Africa [11].

National surveillance and M&E system capacity is a core component of the response to the HIV epidemic as it enables understanding of the evolving nature of the epidemic and the gaps and deficiencies in HIV programs, and it is critical for informing more strategic investments. Capacity development can broadly be classified as pre-service, which is training people before they enter the work force, and in-service, which is training people already in the workforce. In the field of HIV strategic information, pre-service capacity building can take the form of formal courses and tracks in established universities, such as have been developed in M&E at the Mzumbe University in Tanzania, Jimma University in Ethiopia, and the National School of Public Health in Brazil [12-14]. Post-doctoral full-time training programs have also provided substantial practical training opportunities in public health epidemiology through the Centers for Disease Control and Prevention’s Epidemic Intelligence Service and Field Epidemiology and Laboratory Training Programs and more academically focused fellowships, such as those provided through the Fogarty International Center and other institutes of the United States National Institutes of Health [15-18].

Since 2004, the WHO Collaborating Centre for HIV Strategic Information based at the Andrija Stampar School of Public Health at the University of Zagreb in Croatia has been providing in-service capacity-building activities in HIV surveillance and M&E of HIV programs, primarily via training workshops and technical assistance [19]. Twenty-eight training courses in HIV surveillance and M&E of national HIV programs and HIV interventions have been developed with partner institutions. The capacity development activities of the Collaborating Centre target diverse groups that work with and contribute to HIV surveillance, including public health professionals, health care providers, epidemiologists, clinicians, non-governmental organization (NGO) staff, and networks of PLHIV.

The aim of this paper is to describe results of an assessment of capacity development needs in HIV surveillance and M&E in the non-European Union (EU) countries in the WHO EUR.

## Methods

### Assessment of Capacities That Need to Be Developed in HIV Surveillance

To assess the needs for capacity development in HIV surveillance and strategic information systems, we distributed two self-administered questionnaires over email. The first questionnaire was sent to the directors of the national HIV programs of all (N=18) non-EU countries in the WHO EUR. This questionnaire was sent in May 2015 to assess the capacities that needed to be developed in HIV surveillance and addressed the following areas: (1) the types of training courses in HIV surveillance and M&E of HIV programs that national entities organized in 2012, 2013, or 2014 for staff working at the national level; (2) the priority level for strengthening of a range of HIV surveillance and M&E-related areas specified in the questionnaire; (3) the gaps and weaknesses in the areas that respondents marked as being of high or moderate priority for further development; (4) the population groups that might play an essential role in HIV transmission and that have not been covered with surveillance; (5) anticipated impact of declines in funding from the Global Fund to Fight AIDS, Tuberculosis and Malaria (GFATM) on any of the HIV surveillance and M&E activities that have been developed through the support of GFATM.

### Assessment of Availability of Data to Construct HIV Care Cascades

The second, separate questionnaire was sent after the training course in HIV care cascade analysis held in collaboration with WHO in June 2015. This questionnaire was sent to course participants from 15 countries of Eastern Europe and Central Asia (Armenia, Azerbaijan, Belarus, Croatia, Estonia, Georgia, Kazakhstan, Kyrgyzstan, Moldova, Russian Federation, Serbia, Slovenia, Tajikistan, Turkey, and Ukraine) who were invited to the training course by WHO EURO and all worked at the national HIV programs as surveillance professionals. This questionnaire aimed to assess the availability of data to construct cross-sectional and longitudinal HIV care cascade analysis and identify the strengths and weaknesses of cascade indicators [20]. The questionnaire also assessed whether a cross-sectional HIV care cascade can be constructed for key populations, pregnant women, and geographical areas.

## Results

### Capacity Development Needs in HIV Surveillance

Responses to the first questionnaire were received from 10 of 18 countries (Albania, Azerbaijan, FYR Macedonia, Kyrgyzstan, Moldova, Montenegro, Serbia, Tajikistan, Turkey, and Ukraine). In 5 countries out of 10, a national entity had organized training courses in HIV surveillance in 2012, 2013, and 2014. Courses organized were on M&E of HIV programs (mentioned by 4 countries), followed by integrated bio-behavioral surveys (3 countries), key population size estimations (3 countries), using a national HIV database (2 countries), data analysis (2 countries), sentinel surveillance (1 country) and estimation and projection of the HIV epidemic (1 country).

HIV surveillance and M&E-related areas that were most frequently marked as being of high to moderate priority for strengthening were national electronic patient monitoring systems (8 countries), evaluation of HIV interventions and impact analysis (8 countries), HIV care cascade analysis (6 countries), implementation science (5 countries), and data analysis (5 countries). HIV case reporting (7 countries) and developing national HIV surveillance reports (7 countries) were of the lowest priority as these are already developed surveillance components. Among activities of low priority but not yet developed, one country mentioned HIV drug resistance surveillance and another estimation of the number of PLHIV and HIV incidence estimates. Respondents outlined a number of gaps and weaknesses that they felt should be addressed. In relation to HIV case reporting and mortality reporting these were under-estimates of the reported number of HIV-related deaths (7 countries), problems with timeliness and completeness of reporting of HIV cases (7 countries), limited CD4 count testing at the time of HIV diagnosis (4 countries), and lack of electronic reporting of HIV cases (3 countries). Other weaknesses respondents mentioned were lack of HIV drug resistance surveillance (4 countries); lack of capacity in data analysis, interpretation, and use (3 countries); lack of human resources to conduct HIV surveillance in key populations (3 countries); and lack of HIV incidence surveillance (2 countries). Single countries also responded that there was a lack of comprehensive evaluation and impact analysis, lack of capacity for fundraising, limited capacity to implement integrated bio-behavioral surveys and key population size estimates, challenges with estimating the number of PLHIV, and the heavy dependence of HIV surveillance activities on donor funding. Migrant populations, internally displaced persons and refugees and men who have sex with men (MSM) were most commonly mentioned as groups not covered by surveillance (5 countries), followed by clients of sex workers (4 countries). Seven of 8 coutries responded that there will be an impact of declines in funding from GFATM on surveillance, and M&E activities and surveillance in key population reportedly have the lowest sustainability.

### Progress in the HIV Continuum of Care Analysis

Responses to the second questionnaire were received from 13 out of 15 countries (all except Serbia and Slovenia). Tables 1 and 2 show the availability of HIV strategic information, which is needed to construct cross-sectional HIV care cascades and the weaknesses of the data, respectively. In terms of the availability of data to construct a cross-sectional HIV care cascade, 3 countries reported that they were unable to estimate the number of PLHIV, and 4 countries pointed out the need for this indicator but did not provide an explanation on whether they were able to obtain it. The most common weakness in obtaining the number of PLHIV was a lack of trust in modeling outputs and lack of high quality input data necessary for modeling tools to provide reliable results.

**Table 1:** Availability of HIV (human immunodeficiency virus) strategic information needed to construct cross-sectional HIV care cascades (n=13).

Type of indicator	Number of countries where available (n)	No response (n)
Estimated number of people living with HIV (human immunodeficiency virus)^a^	5	1
Number of people diagnosed with HIV who know their status	10	3
Number of PLHIV^b^ who received HIV care in the past 12 months, including ART^c^	9	4
Number of PLHIV currently receiving ART	9	4
Number of PLHIV currently receiving ART who have a suppressed viral load (<1000 copies/mL)	9	4

^a^Four countries reported that this indicator is needed but did not specify whether they were able to obtain it, and three reported that the indicator is not available.

^b^PLHIV: people living with HIV.

^c^ART: antiretroviral treatment.

**Table 2:** Most frequently reported weaknesses of data and data sources used to construct cross-sectional HIV (human immunodeficiency virus) care cascades (n=13)

Indicator	Weakness	Number of countries
Estimated number of people living with HIV (human immunodeficiency virus)	A lack of good quality input data is necessary for modeling, which leads to low quality modeling outputs	3
	Used Spectrum^c^ modeling tool but would like to use an additional tool (lack of trust in Spectrum estimates)	2
	Lack of trust in the modeling estimates	2
Number of people diagnosed with HIV who know their status	Poor mortality statistics: mortality statistics are not linked with HIV case reporting system	6
Number of PLHIV^a^ who received HIV care in the past 12 months, including ART^b^	There is a lack of linkage of patient monitoring system with the HIV case reporting system	2
	It is challenging to ensure the quality of data at the local level	2
Number of PLHIV currently receiving ART	Lack of disaggregation by ART regimens and key populations	2
Number of PLHIV currently receiving ART who have a suppressed viral load (<1000 copies/mL)	Limited access to viral load diagnostics	4

^a^PLHIV: people living with HIV.

^b^ART: antiretroviral treatment.

^c^Specturm: analytical tool used for mathematical modeling of HIV epidemics used by UNAIDS

The majority of the countries (n=10) have data on the number of people diagnosed with HIV who know their HIV status. The most common reported weakness of these data was poor quality mortality statistics, which makes it difficult to assess the cumulative number of people living with diagnosed HIV. In addition, multiple registrations of newly diagnosed HIV cases was mentioned by one country and under-reporting of newly diagnosed cases (due to not obtaining HIV positive test result when people tested for HIV) by another country.

Nine countries reported that they were able to provide the number of PLHIV who received HIV care in the past 12 months, including ART. Alongside the shortcomings shown in Table 2, an inability to record ART interruptions in the patient monitoring system was reported by one country and a lack of clear criteria for reporting on patients enrolled in HIV care but not on ART by another country.

Nine countries reported that they were able to provide the number of PLHIV who are currently on ART, and the availability of a centralized HIV care and treatment database was mentioned by 3 countries as one of the major advantages in obtaining this indicator. The most commonly mentioned deficiency was limited disaggregation of data by ART regimens and key populations.

Nine countries reported that they were able to report on the number of PLHIV currently receiving ART who have a suppressed viral load (<1000 copies/mL). As a weakness, 3 countries indicated limited access to viral load diagnostics, while other challenges reported included a non-standardized definition of viral suppression at a national level (1 country) and a lack of an information system for collecting data on HIV viral load (1 country).

With regard to disaggregation of the HIV care cascade, only 3 countries out of 13 reported that they were able to construct a cascade for each of the key populations: people who inject drugs (PWID), MSM, and female sex workers (FSW). Eleven countries reported that the cascade can be created for PWID, 10 for MSM, and 3 for FSW. One country reported that the disaggregation was not possible, and three reported that geographical disaggregation into sub-national units was not possible. However, disaggregation for pregnant women was possible in 9 countries.

Twelve countries reported the availability of a longitudinal HIV patient monitoring system. Six countries reported that they could calculate the percentage of people diagnosed with HIV who were enrolled in HIV care within 12 months of HIV diagnosis. The same 6 countries reported that they were able to calculate the proportion of people diagnosed with HIV and on ART who were retained on ART for at least 12 months and have a suppressed viral load. They were also able to report the percentage of HIV-exposed infants who received a virological test within two months of birth and the proportion of HIV-exposed infants who were uninfected after cessation of breastfeeding.

## Discussion

### Principal Findings

We identified a diverse set of needs for capacity development in HIV strategic information among the countries that participated in the assessment. The greatest development needs were reported in the areas of national electronic patient monitoring systems, evaluation of HIV interventions and impact analysis, implementation science, HIV care cascade analysis, and data analysis. Of note is that, in the context of declines in funding for HIV surveillance, M&E activities and surveillance in key populations will have, as reported by the national HIV program staff, the lowest sustainability.

The countries reported a complex list of capacities, and six appeared to have very functional data systems able to report most aspects of the HIV treatment cascade. There was, however, substantially less ability to disaggregate data geographically or by key population, a capacity of great importance in a region where the most HIV epidemics are concentrated.

A common weakness was poor quality of mortality statistics, which limits the ability to assess how many PLHIV are diagnosed. In addition, although the majority of surveyed countries have longitudinal HIV patient monitoring systems, only one-half could report on the proportion of patients who have a suppressed viral load 12 months after ART initiation. Understanding longer-term outcomes of the HIV continuum of care such as retention of treatment and viral suppression should be enabled through investment in infrastructure and human capacities with the aim to support national and global efforts to monitor progress towards the 90-90-90 targets [4].

This assessment points to the need to further invest in capacities in order to develop HIV strategic information systems that will provide necessary data for evidence-based decision making and for evaluating the access, coverage, and quality of interventions. A recent review identified several factors that drove the success in HIV treatment monitoring globally in the period from 2000-2015 [21]. These were commitment to invest in country data systems, aiming for 5-10% of program funds to be used to strengthen M&E, creating more demand for the data by conducting regular country program and epidemiological reviews, which were subsequently used as a basis for seeking funding from the donor agencies, and setting program targets [21].

Ability to report on some of the key impact indicators of HIV programs in Eastern Europe and Central Asia —viral load suppression and mortality—–should be substantially strengthened, considering that the rates of viral load suppression among PLHIV are among the lowest globally [9,11]. Having in place uniform reporting practices and providing standardized guidelines and evaluation tools to surveillance programs were identified as policies that can increase effectiveness of surveillance, along with maintaining relationships with providers and laboratories to ensure complete and timely reporting [22]. Ongoing support from stakeholders to sites that provide HIV care and treatment and on-site training was also found to be important, particularly in settings where HIV treatment is being scaled-up and decentralized and patient monitoring systems established [23]. Timely provision of periodic reports from treatment sites also assisted in informing program management on the quality of patient care and enabled actions to be taken to improve treatment outcomes [23,24].

### Limitations

Our data are subject to limitations. A few countries we attempted to survey did not participate, and we did not conduct site visits to verify reported capacity. Results of the assessment are applicable to the countries that responded to the questionnaires. Additionally, responses to some questions were missing. Nonetheless, we believe that our data will be helpful in planning the next round of strategic information capacity building in the region.

## Abbreviations

ART: antiretroviral treatment
HIV: human immunodeficiency virus
NGO: non-governmental organization
PLHIV: people living with HIV
